# Development and validation of a Japanese version of the person-centered primary care measure

**DOI:** 10.1186/s12875-022-01726-7

**Published:** 2022-05-10

**Authors:** Makoto Kaneko, Tadao Okada, Takuya Aoki, Machiko Inoue, Takamasa Watanabe, Makoto Kuroki, Daichi Hayashi, Masato Matsushima

**Affiliations:** 1 Department of Health Data Science, Yokohama City University, 22–2, Seto, Kanazawa-Ku, Yokohama, Kanagawa 236–0027 Japan; 2 Tessyokai Kameda Family Clinic Tateyama, Chiba, Japan; 3 Division of Clinical Epidemiology, Research Center for Medical Sciences, The Jikei University School of Medicine, Tokyo, Japan; 4 Department of Family and Community Medicine, Hamamatsu University School of Medicine, Shizuoka, Japan; 5 Centre for Family Medicine Development, Japanese Health and Welfare Co-operative Federation, Tokyo, Japan; 6 Department of Radiology, Stony Brook University Renaissance School of Medicine, Stony Brook, NY 11790 USA

**Keywords:** Family medicine, Patient-centered care, Patient experience, Primary care assessment tool, Primary health care, Quality measurement

## Abstract

**Background:**

Although primary care (PC) is an indispensable part of the health system, measuring its quality is challenging. A recent measure of PC, Person-Centered Primary Care Measure (PCPCM), covers 11 important domains of PC and has been translated into 28 languages. This study aimed to develop a Japanese version of the PCPCM and assess its reliability and validity.

**Methods:**

We employed a cross-sectional mail survey to examine the reliability and content, structure, criterion-related, and convergent validity of the Japanese version of the PCPCM. This study targeted 1000 potential participants aged 20–74 years, selected by simple random sampling in an urban area in Japan. We examined internal consistency, confirmatory factor analysis, correlation between the Japanese version of the Primary Care Assessment Tool-Short Form (JPCAT-SF), and the association between the PCPCM score and influenza vaccine uptake.

**Results:**

A total of 417 individuals responded to the survey (response rate = 41.7%), and we used the data of 244 participants who had the usual source of care to assess the reliability and validity of the PCPCM. Confirmatory factor analysis demonstrated sufficient structural validity of the original one-factor structure. The overall Cronbach’s alpha was 0.94. The Spearman correlation coefficient between PCPCM and JPCAT-SF was 0.60. Influenza vaccine uptake was not significantly associated with total PCPCM score.

**Conclusions:**

The study showed that the Japanese version of the PCPCM has sufficient internal consistency reliability and structural- and criterion-related validity. The measure can be used to compare the quality of primary care in Japan and other countries.

## Background

Primary care (PC) is an important part of the healthcare system because it is associated with the effective use of healthcare resources and health equity [1–4]. PC contributes to not only disease-specific care but also the overall performance of healthcare systems through the reduction of inequity, patient-centeredness, access, continuity, coordination, comprehensiveness, quality, and efficiency of care [5]. Due to its nature, measuring the quality of PC is challenging [6]. Disease-specific measures cannot assess indispensable domains of PC as above [7, 8]. In contrast, comprehensive measures including many aspects of PC require a long list of questions, and such measures are not suitable for use in daily practice [9].

To overcome these problems, Etz et al. developed in the United States the Person-Centered Primary Care Measure (PCPCM) in 2019 [9]. PCPCM covers 11 important domains of PC: accessibility, comprehensiveness, integration, coordination, relationship, continuity, advocacy, family context, community context, goal-oriented care and health promotion [9]. PCPCM consists of 11 questions and each question corresponds to each domain [9]. The strength of PCPCM is that it was developed based on PC quality indicators selected in a crowd-sourced survey that recruited a large group of Internet-based volunteers including patients, clinicians and purchasers of health care plans [9]. Moreover, the indicators were refined and revised by multidisciplinary healthcare providers, international PC leaders, insurers, patients, policy makers and professional association leaders [9]. The PCPCM focus on not only the quality of care but also the complexity of primary care to address a patient as a whole person [5]. In particular, the items such as integration, relationship, family context and community context have addressed person-centerdness [10]. To assess individualized, person-centered care from the patient’s perspective is necessary to accomplish generalist practice [11]. Additionally, the reliability and validity of PCPCM were assessed through online and clinical samples [9]. Since its development, PCPCM has been translated into 28 languages [12], and has been reported to be “robust and capable of capturing the essential functions through which PC operates” [5].

In Japan, the Japanese version of the Primary Care Assessment Tool (JPCAT) has been widely used to assess the quality of PC [13–16]. JPCAT includes 29 items and six domains [13]: first contact, longitudinality, coordination, comprehensiveness and community orientation. The JPCAT-Short Form (SF) consists of 13 items and 6 domains [17]. Importantly, because JPCAT was developed by modifying the original version of the Primary Care Assessment Tool in the US [18] to fit the Japanese context, the score of the JPCAT cannot be compared with that of the original tool directly. Since the PCPCM can assess the broader components of PC quality with fewer items and person-centeredness from the patient’s perspective, the development of a Japanese version of the PCPCM may be useful in identifying the strengths and weaknesses of PC in Japan compared to other countries.

Therefore, this study aimed to develop a Japanese version of the PCPCM and assess its reliability, content, structural, criterion-related, and convergent validity.

## Methods

### Study design, setting, and population

We conducted a cross-sectional study using a mail survey to assess the reliability and validity of the Japanese version of the PCPCM. Reliability was evaluated by internal consistency, and the validity assessment included content, structural, criterion-related, and convergent validity.

Using simple random sampling, we selected 1000 potential participants aged 20–74 years using a basic resident register. The sampling site was Konan-ku, located in the city of Yokohama, Japan, because this area has a similar proportion of people aged 65 years and older (28.6%), and people who receive government safety net programs for livelihood, housing, and healthcare services (1.5%) compared to overall Japan [19]. Yokohama is located next to Tokyo and is the most populated basic municipality in Japan.

The participants answered questions about the Japanese version of the PCPCM, JPCAT-SF, age, sex, years of education, household income, self-rated health, number of years the patient had been with the physician, number of years the patient had been with the practice, and influenza vaccination status. Data were collected between August and September of 2021. Regardless of whether the recipients of the survey responded, the participants also received small gifts worth 500 JPY (approximately 5 US dollars).

### Development of the Japanese version of the PCPCM

In accordance with the Principles of Good Practice for the Translation and Cultural Adaptation Process for Patient-Reported Outcomes Measures [20], we developed the Japanese version of the PCPCM. First, the 11-item PCPCM was translated into Japanese by one of the authors (MK) and the validity and clarity of the content and adjustment of the phrasing of the Japanese version were evaluated by our research team. The research team included experts of primary care in Japan such as five certified family physicians and a primary care researcher. The experts discussed not only the expressions or meanings in the Japanese version but also the relevance and comprehensiveness for patients in a primary care setting in Japan. A back translation (Japanese to English) was conducted by a bilingual physician (a co-author, DH) who practiced medicine in both English and Japanese languages and was not familiar with the original English version of the questionnaire. The back translation was sent to the original PCPCM team in the United States and they did not indicate a significant change of meanings from the original version. The Japanese version was revised based on the feedback, mainly about wording or expression. Another round of back translation was subsequently performed and both teams reached a consensus. Finally, we conducted cognitive debriefing to assess the cognitive equivalence of the Japanese version [20]. Interviews were conducted with seven patients (two males and five females) in a family medicine clinic in Japan. We targeted the patients who visited the clinic for regular consultation and the median age was 66 (interquartile range: 35-83). Regarding their academic background, two of them graduated from a junior high school, three from a high school and others completed a college or a university. The interviewer conducted semi-structured interviews. The guide questions based on the COSMIN Study Design checklist [21] are as follows: 1) What do you think about assessing patient experience through the PCPCM (relevance). 2) Do you have additional aspects of primary care which are not covered by the PCPCM (comprehensiveness). 3) Please let me know if you have any difficulties understanding the questionnaire (comprehensibility).4) If you have any comments, please let me know. As the result, all participants did not feel any difficulty understanding the Japanese version of the PCPCM and there were no additional aspects or factors to add to the PCPCM. However, four participants felt the term “a minority group” was difficult to understand. Therefore, our team added the example in the Japanese version: a minority group (such as an ethnic minority or sexual minority). The discussion among experts and cognitive debriefing ensured the content validity of the Japanese version of the PCPCM [22, 23]. The Japanese version of the PCPCM and the fielding kit are available from the authors and they are also available on the original PCPCM website by The Larry A. Green Center: https://www.green-center.org/pcpcm.

As with the original PCPCM, the Japanese version consists of 11 questions: accessibility, comprehensiveness, integration, coordination, relationship, continuity, advocacy, family context, community context, goal-oriented care, and health promotion [9]. Each item is given a 1–4-point (maximum 4 points) rating. The total score of the PCPCM was calculated as the mean of the score of all items.

Because the role of general practitioner/family physicians is ambiguous in Japan [24], patients or residents might find it difficult to answer questions such as “How would you assess your primary care experience?” or, “My practice makes it easy for me to get care.” Therefore, we targeted participants who had the “usual source of care (USC)” as the respondents of the questionnaire. We used the question, “Is there a medical facility to whom you usually go if you are sick or need advice about your health?” to identify whether the participant had USC [18]. This was done because the JPCAT-SF employs a similar question for the same purpose [17], namely “Is there a doctor to whom you usually go if you are sick or need advice about your health?” Thus, in the current study, we employed the former question for the PCPCM and the latter question for the JPCAT-SF.

### Reliability and validity

We examined the psychometric properties of the Japanese version of the PCPCM using the following steps based on the COSMIN Study Design checklist for patient-reported outcome measurement instruments [21]. First, internal consistency reliability was assessed using item-total correlations and Cronbach’s α reliability coefficients. Item-total correlation exceeding 0.30 and alpha value ≥0.8 was recommended [25]. Second, regarding content validity and translation process, we interviewed patients about the relevance, comprehensiveness, and comprehensibility of the Japanese version of the PCPCM. Also, the experts discussed the relevance and comprehensiveness of the PCPCM. Third, to examine structural validity, we conducted a confirmatory factor analysis because we hypothesised the same one-factor structure as that of the original PCPCM. We employed diagonally weighted least squares for categorical factor analysis of categorical data. The appropriateness of the resulting structure was determined based on factor loadings. Factor loadings ≥0.4 were employed as an indicator [26]. Model fitness was evaluated based on the comparative fit index (CFI), Tucker-Lewis index (TLI), root mean square error of approximation (RMSEA), and standardised root mean square residual (SRMR). For CFI and TLI, values > 0.95 indicated goodness of fit. In addition, RMSEA < 0.06, and SRMR < 0.08 were employed as representative of models with good fit [27]. Forth, criterion-related validity was examined by correlating the total score of the PCPCM with the total score of the JPCAT-SF [17]. We used the JPCAT-SF because the measure of patient experience is often used in the Japanese PC setting and associations between the JPCAT-SF and patient outcomes, such as vaccine uptake [28] or cancer screening [14], have been demonstrated. Fifth, convergent validity was assessed using hypothesis testing. Some studies have reported an association between high patient experience scores and preventive care in the PC setting [28–30]. In the current study, our hypothesis was that a high PCPCM total score would be positively associated with influenza vaccine uptake. We categorised the PCPCM total score into quartiles and examined the associations of this score with influenza vaccine uptake using the Cochran–Armitage trend test. We also examined the association between influenza vaccine uptake within the past year [28] and the total and item-specific score of the PCPCM. We employed a logistic regression model to adjust for age, sex, years of education, household income, and self-rated health [28, 31, 32]. We included age and PCPCM scores as continuous variables and sex, household income, education, and self-rated health as categorical variables in the model. In addition, we calculated the odds ratio (OR) per 1 standard deviation (SD) increase in the PCPCM total score to influenza vaccine uptake. We did not evaluate measurement errors such as test-retest validity and responsiveness because of difficultiy of conducting follow-up survey. We also conducted descriptive statistics (mean, SD, median, interquartile range, and percentage) for the PCPCM and the demographics of the participants. In logistic regression, we used multiple imputations for missing values by fully conditional specification, which included age, sex, years of education, household income, self-rated health, and vaccination status. In the confirmatory factor analysis, we employed full information maximum likelihood for missing variables. The scores of the PCPCM and JPCA-SF were calculated based on each regulation. All statistical analyses were conducted using StataCorp. 2017. Stata Statistical Software: Release 15. College Station, TX: StataCorp LLC and R version 4.1.1 (R Foundation for Statistical Computing, Vienna, Austria; https://www.r-project.org).

To examine the properties of the PCPCM and perform a satisfactory factor analysis, we estimated a minimal sample size of 200 residents who have USC [21, 33]. Based on our estimated response rate of 20–30% and the previously reported proportion of individuals who had USC in Japan (50%) [13], we sent the questionnaire to 1000 residents.

## Results

A total of 417 individuals responded to the survey (response rate = 41.7%). However, 14 were excluded by a blank ballot; thus, the study was conducted on the remaining 403 respondents. The respondents were older than the non-respondents (mean ages: 51.8 vs. 46.5, respectively), and men were less likely to participate in the survey than women (male proportions: 43.2% vs. 55.7%, respectively). Of the 403 individuals, 244 (60.5%) had USC. Table 1 shows the demographics of the individuals with and without USC. The mean total score of the PCPCM was 2.59, the SD was 0.71, and the standard error was 0.003. Table 2 presents the descriptive features of the PCPCM.

**Table 1:** Demographics of the participants (*n* = 400, missing information on usual source of care = 3)

	With USC *n* = 244: n (%)	Without USC *n* = 156: n (%)
Sex		
Male	109 (44.7)	64 (41)
Female	134 (54.9)	92 (59)
Others	1 (0.4)	0
No response	0	0
Age (year)		
20–29	16 (6.6)	12 (7.7)
30–39	31 (12.7)	32 (20.5)
40–49	43 (17.6)	32 (20.5)
50–59	57 (23.4)	38 (24.4)
60–69	55 (22.5)	32 (20.5)
70–74	42 (17.2)	10 (6.4)
No response	0	0
Education		
Less than high school	8 (3.3)	1 (0.6)
High School	52 (21.3)	39 (25)
Junior College	64 (26.2)	37 (23.7)
More than or equal to college	119 (48.8)	79 (50.6)
No response	1 (0.4)	0
Annual household income (million JPY)		
< 2.00 (=18,000 US dollar)	33 (13.5)	6 (3.8)
2.00–5.00	92 (37.7)	55 (35.3)
≥ 5.00	116 (47.5)	94 (60.3)
No response	3 (1.2)	1 (0.6)
Self-rated health status		
Excellent	9 (3.7)	17 (10.9)
Very good	23 (9.4)	18 (11.5)
Good	59 (24.2)	36 (23.1)
Poor	108 (44.3)	81 (51.9)
Very poor	44 (18)	4 (2.6)
No response	1 (0.4)	0
Minority group		
Yes	14 (5.7)	6 (3.8)
No	230 (94.3)	150 (96.2)
No response	0	0
How many years have you known this doctor?		
< 1	1 (0.4)	Not applicable
1–9	106 (43.4)	
10–20	87 (35.7)	
20≥	46 (18.9)	
missing	4 (1.6)	
How many years have you known this practice?		
< 1	0	Not applicable
1–9	105 (43.0)	
10–20	86 (35.2)	
20≥	53 (21.7)	
missing	0	
Types of USC		
Clinic	213 (87.3)	
Hospital	29 (11.9)	
Both	1 (0.4)	
Missing	1 (0.4)	

*USC* usual source of care

**Table 2:** Descriptive feature of the new Japanese version of the PCPCM (*n* = 243)

Domain (n)	Mean score	Standard deviation	25th percentile	50th percentile	75th percentile	Observed range	Skewness	Kurtosis
PCPCM								
Total score (243)	2.59	0.71	2	2.64	3.09	1.09–4	0.11	2.33
Accessibility: The practice makes it easy for me to get care (241)	2.98	0.78	2	3	4	1–4	−0.23	2.32
Comprehensiveness: This practice is able to provide most of my care (243)	2.91	0.80	2	3	3	1–4	−0.23	2.38
Integration: In caring for me, my doctor considers all factors that affect my health (242)	2.66	0.92	2	3	3	1–4	−0.09	2.16
Coordination: My practice coordinates the care I get from multiple places (240)	2.54	0.94	2	2.5	4	1–4	0.03	2.10
Relationship: This doctor or practice knows me as a person (243)	3.16	0.86	2	3	4	1–4	−0.05	2.03
Continuity: My doctor and I have been through a lot together (241)	2.14	0.92	1	2	3	1–4	0.48	2.42
Advocacy: My doctor or practice stands up for me (242)	2.48	0.86	2	2	3	1–4	0.20	2.37
Family context: The care I get takes into account knowledge of my family (238)	2.18	0.98	1	2	3	1–4	0.46	2.23
Community context: The care I get in this practice is informed by knowledge of my community (239)	2.12	0.95	1	2	3	1–4	0.48	2.33
Goal-oriented: Over time, this practice helps me to meet my goals (242)	2.57	0.91	2	3	3	1–4	0.02	2.17
Health promotion: Over time, my practice helps me stay healthy	2.68	0.92	2	3	3	1–4	−0.1	2.13

*PCPCM* Person-Centered Primary Care Measure

### Construct, criterion-related and convergent validity

**Fig. 1:**
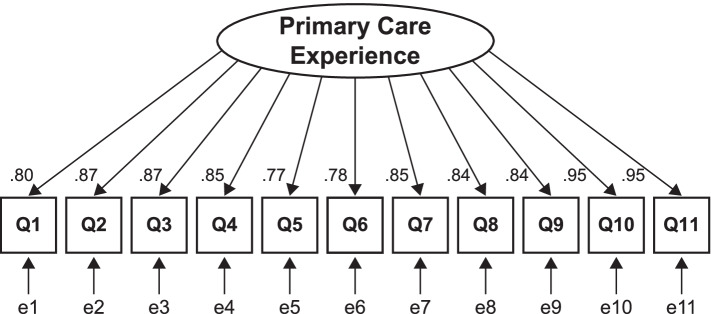
Factor structure of the Japanese version of the Person-Centered Primary Care Measure (confirmatory factor analysis). Squares are observed variables (items); ellipses are latent variables (factors), values on the single-headed arrows are standardised factor loadings

**Fig. 2:**
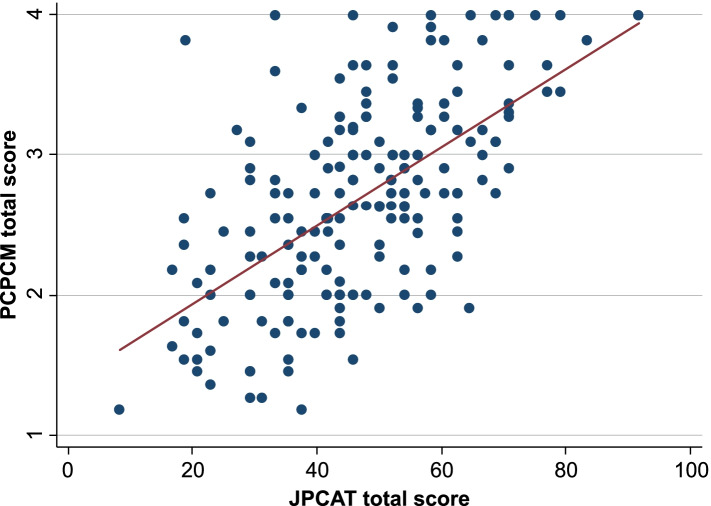
Scatter plot of the Japanese version of the Person-Centered Primary Care Measure and the Japanese version of Primary Care Assessment Tool-Short Form

**Fig. 3:**
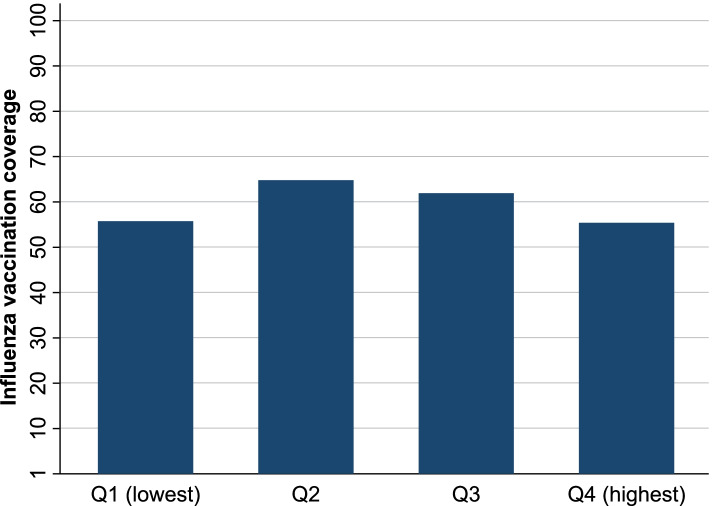
Associations of the Japanese version of the Person-Centered Primary Care Measure total score with influenza vaccination

Figure 1 shows the path diagrams of the confirmatory factor analysis used to assess the structural validity of the one-factor structure of the Japanese PCPCM. All factor loadings of each item on each factor were ≥ 0.40. Confirmatory factor analysis showed a sufficient fit (CFI = 0.991, TLI = 0.989, RMSEA = 0.159, and SRMR = 0.077). Regarding criterion-related validity, the correlation coefficient γ between the PCPCM total score and the JPCAT-SF total score was 0.60 (Fig. 2). In terms of convergent validity, the overall vaccine coverage among participants with USC was 58.9%. The associations between PCPCM total score and influenza vaccination coverage are presented in Fig. 3. In the Cochran-Armitage trend test, the PCPCM total score was not significantly associated with influenza vaccine uptake (*p* = 0.8626). Moreover, in the logistic regression analysis, the PCPCM total score was not statistically significantly associated with influenza vaccine uptake: OR per 1SD was 1.19 (95% confidence interval [CI]: 0.91–1.56, *p* = 0.22). However, coordination and advocacy were positively associated with vaccine uptake: OR per 1SD 1.37 (95%CI: 1.01–1.78, *p* = 0.04), OR 1.40 (95%CI: 1.05–1.87, *p* = 0.02), respectively. In the same dataset, the JPCAT-SF total score was also not associated with vaccine uptake: OR 1.02 (95%CI: 1.00–1.04, *p* = 0.088). Table 3 shows OR per 1SD of the PCPCM total and item-specific score.

**Table 3:** Associations of the PCPCM score with influenza vaccination (*n* = 243)

	Adjusted Odds Ratio (95% Confidence Interval)	*p*-value
PCPCM		
Total score	1.19 (0.91–1.56)	0.22
Accessibility	0.99 (0.75–1.35)	0.91
Comprehensiveness	1.07 (0.82–1.4)	0.61
Integration	1.08 (0.82–1.42)	0.59
Coordination	1.34 (1.01–1.78)	0.04
Relationship	1.16 (0.89–1.51)	0.28
Continuity	1.12 (0.85–1.47)	0.44
Advocacy	1.40 (1.05–1.87)	0.02
Family context	1.18 (0.89–1.55)	0.25
Community context	0.99 (0.75–1.32)	0.96
Goal-oriented	1.23 (0.94–1.63)	0.13
Health promotion	1.11 (0.84–1.44)	0.48

*PCPCM* Person-Centered Primary Care Measure

### Reliability of internal consistency

The overall Cronbach’s alpha coefficient of the PCPCM was 0.94, and the Cronbach’s alpha coefficient for all items was > 0.90. The item-total correlation ranged from 0.66 –0.80.

## Discussion

In the present study, we developed a Japanese version of PCPCPM that showed sufficient internal consistency, reliability, and structural and criterion-related validity. In terms of convergent validity, vaccine uptake was not significantly associated with the total PCPCM score. However, the PCPCM had sufficient reliability and validity for assessing patient experience in PC in the Japanese setting.

Zyzanski et al. Examined the psychometric properties of the Japanese version of the PCPCM in 35 The Organisation for Economic Co-operation and Development (OECD) countries, including Japan [12]. However, their study had several limitations. First, regarding the validity, Zyzanski et al. only assessed criterion-related validity and did not examine other types of validity such as convergent validity and structural validity. Second, the translation of English to other languages was conducted by a single independent global company [12]. Therefore, contextual factors such as culture or healthcare systems could not be fully considered. Moreover, whether the company and research team included a native speaker or bilingual member was unclear. In Japan, respondents who answered “no” to the question, “Was it hard to complete this form?”, had higher PCPCM scores than those who answered “yes” [12]. The result may have demonstrated the respondents’ insufficient understanding of the questionnaire. Third, terms and phrases about PC in PCPCM are difficult for Japanese participants to understand. In Japan, because there is no gate-keeping system by a PC physician and a patient can access secondary care directly [24, 34], respondents might not understand some phrases in the PCPCM, such as: “your primary care experience” in “How would you assess your primary care experience?” and “my practice” in “My practice makes it easy for me to get care.” To overcome these limitations, we, a team of Japanese family physicians, developed the alternative Japanese version of the PCPCM, focusing on participants who have USC.

Regarding the results of confirmatory factor analysis, RMSEA did not meet the criteria set a priori. However, other evaluation standards fulfilled the criteria. Therefore, we regarded the structural validity as sufficient. In criterion-related validity, the correlation coefficient between the score of the PCPCM and the JPCAT was the border between moderate and strong [35]. This might mean the PCPCM and the JPCAT did not necessarily measure the exact same thing. The PCPCM focuses on the assessment of person-centeredness compared with the JPCAT and it is the characteristics of the measure [5].

In terms of the PCPCM score, the mean total score in our study was 2.59. This was slightly higher than Zyzanski et al.’s reported score of 2.46 [12]. A possible reason is that the participants in our study had USC and they answered the questionnaire with their USC in mind. Furthermore, our translation might be understandable for participants who are native Japanese speakers. The mean PCPCM score of 2.59 obtained in our study was, however, not a good result compared to other OECD countries [12]. If the score in the current study adapted the rank of the PCPCM total score of the previous study, Japan would rank 28th among 35 OECD countries [12]. This suggests that there is room for improvement in Japanese PC. In terms of each item, continuity, family context, and community context had relatively low scores of 2.14, 2.18, and 2.12, respectively. The scores of these domains were also the worst-three domains in a previous study in the US, both in online and clinical sampling [9]. Therefore, these domains may reflect the common challenges of PC in Japan and the US.

Although our version of the Japanese PCPCM showed sufficient internal consistency reliability, criterion-related validity, and construct validity, convergent validity demonstrated inconsistent results when compared to previous studies that used other patient experience measures [17, 28]. Even in the original PCPCM, the score and outcome of patient care have not been examined. Future studies should, therefore, be conducted to assess the association between PCPCM scores and patient outcomes.

### Implications of the study

The PCPCM can assess various aspects of PC and compare the results with those of other countries. The PCPCM has been translated into 28 languages and has assessed reliability and validity in 35 OECD countries [12]. Therefore, our version of the PCPCM is useful for detecting the strong and weak points of Japanese PC compared to other countries. The comparison may ultimately help policymakers, PC physicians, and residents to improve the quality of PC in Japan. Moreover, the PCPCM can also assess person-centeredness from the patient perspectives which is vital for generalist practice [11]. This is an important step to foster person-centered primary care in Japan [11, 36].

### Study strengths

Our version of the Japanese PCPCM was developed by a research team that included native Japanese PC physicians, PC researchers, and a bilingual physician who practiced medicine in Japan, the United Kingdom, and the United States, which This is useful for creating an understandable measure for the Japanese native population. Moreover, because this study employed a mail survey and recruited randomly selected adults from the general population, compared to the previous Japanese version, the results of our study could be generalised to people in other areas of Japan. In addition to the previous Japanese version by Etz et al. [9], this study assessed the convergent validity of the PCPCM in the Japanese setting; these results might be helpful in interpreting the meaning of the PCPCM in Japan.

### Study limitations

Our study has several potential limitations. First, measurement errors such as test-retest reliability and responsiveness were not assessed because of difficultiy of conducting follow-up survey, and convergent validity and discriminant validity were not confirmed by hypothesis testing. Second, non-response bias may have affected the results. Individuals with negative past PC experiences might not have responded to the survey. Therefore, respondents might have given a higher score than non-respondents, and patient experience in Japan may have been overestimated. Third, our study was conducted in an urban area. Although we selected the study area based on the proportion of the older population and people who received public assistance, other factors were not considered. Thus, the results need to be interpreted carefully. A survey on the PCPCM in other settings such as a rural area should, therefore, be conducted in the future.

## Conclusions

The alternative version of the Japanese PCPCM showed sufficient reliability and validity. Our version was created specifically for Japanese patients by Japanese physicians/researchers and can be used to compare the quality of PC in Japan and in other countries. Understanding the strengths and weaknesses of PC in Japan is important for effective PC development.

## Data Availability

The datasets generated during and analysed during the current study are not publicly available because we did not receive informed consent concerning data sharing from the participants.But the datasets are available from the corresponding author on reasonable request.

## Abbreviations

PCPCM: Person-Centered Primary Care Measure
JPCAT: Japanese version of the Primary Care Assessment Tool
JPCAT-SF: Japanese version of the Primary Care Assessment Tool Short Form
PC: Primary care
OECD: Organisation for Economic Co-operation and Development
CFI: Comparative fit index
TLI: Tucker-Lewis index
RMSEA: root mean square error of approximation
SRMR: Standardised root mean square residual
